# Speed‐Dependent Interjoint Coordination During Treadmill Running in a Poststroke Athlete: A Case Report

**DOI:** 10.1002/ccr3.71354

**Published:** 2025-10-25

**Authors:** Noboru Chiba, Tadayoshi Minamisawa

**Affiliations:** ^1^ Department of Occupational Therapy Yamagata Prefectural University of Health Sciences Yamagata Japan; ^2^ Department of Physical Therapy Yamagata Prefectural University of Health Sciences Yamagata Japan

**Keywords:** ankle joint, biomechanical phenomena, gait analysis, range of motion, running, stroke

## Abstract

In a high‐functioning poststroke runner, the paretic limb shows rigid distal coupling, while the nonparetic limb adapts with speed; at ≥ 6 km/h, hip–ankle timing reverses (ankle leads). Prioritize distal push‐off and interjoint timing retraining to improve efficiency.

## 1 Introduction

Running after a stroke is uncommon because hemiparesis, spasticity, and interjoint discoordination usually constrain high‐speed locomotion. Nonetheless, a minority of survivors regain running capacity, offering a unique window into compensatory strategies and performance‐oriented rehabilitation. Prior biomechanical literature on parasports has emphasized amputee running, muscle‐work distribution, joint moment characteristics, and device‐related loading, while stroke‐related running remains underdescribed [1, 2, 3, 4, 5, 6]. Speed strongly modulates stiffness and joint kinetics in able‐bodied running [7, 8], and treadmill kinematics are broadly comparable to overground kinematics, enabling controlled speed manipulation [9]. Building on our preliminary overground report on the same athlete [10], the present case integrates overground mechanics with treadmill multi‐speed coordination metrics, aiming to clarify speed‐dependent interjoint coordination and joint‐specific compensation with clinical implications for reintegration into parasports [11].

This submission extends our prior overground case [10] by introducing multi‐speed treadmill analyses, continuous Lissajous coupling, and cross‐correlation–based timing lags. These elements and all figures/text are original to this report.

### 1.1 Patient Information

A male poststroke athlete in his 40s, 6 years after right hemiplegia (Brunnstrom stage V in upper and lower limbs), was an independent community ambulator without an orthosis and was licensed to drive. Height: 167 cm; weight: 57 kg (body mass index: 20.4 kg/m^2^). He was taking antihypertensive medication, had completed 2 years of rehabilitation, had returned to home living, and started new employment, and had not received ongoing outpatient therapy. He is currently training in parasports (swimming, running, and cycling) to improve his competitive performance. Written informed consent included permission for publication of this report. The study protocol was approved by the Institutional Ethics Committee of Yamagata Prefectural University of Health Sciences (Approval no. 1501‐14). This participant was included in our previous overground case report; however, the multi‐speed treadmill protocol and temporal coordination analyses (continuous Lissajous coupling and cross‐correlation‐based timing lags) are novel to the present work. The historical and current information regarding this episode of care is summarized in Table 1.

**TABLE 1:** Timeline—Historical and current information from this episode of care organized as a timeline.

Date/phase	Context/setting	Key events/findings	Actions/interventions	Outcome/notes
Y–6	Acute stroke onset, right hemiplegia	Rehabilitation initiated	Inpatient/outpatient rehabilitation	Functional recovery progressed over the next 2 years.
Y–4	Postrehabilitation, community setting	Completed 2‐year rehab, independent outdoor mobility, on antihypertensive medication, no ongoing outpatient rehab	Home/community exercise as tolerated, medication management	Maintained independence in daily mobility
Y–1–present	Para‐sport participation	Began swimming/running/cycling; training aimed at performance improvement	Self‐directed/club training toward competitive goals	Increased training load; candidate for instrumented gait assessment
Y–0 (prep)	Preassessment screening	Safety screening and treadmill familiarization	Prespecified discontinuation criteria, harness/handrails and emergency stop available	Cleared for protocol, no adverse events during familiarization
Y–0 (study)	Laboratory treadmill; 3/4/6/8 km/h, 60 s each	Continuous Lissajous coupling and CCF‐derived timing lags assessed; speed‐dependent inversion of hip–ankle timing (ankle leads ≥ 6 km/h)	Kinematic data capture and analysis	Actionable targets identified: distal push‐off strengthening; timing retraining (lag ≈0.06–0.10 s at higher speeds)
Follow‐up (ongoing)	Clinic and training	Sport‐specific training continued, monitoring of running tolerance and goals	Distal push‐off strengthening, timing‐focused gait retraining using metronome and graded speed progression.	Periodic clinic follow‐up planned

Abbreviation: CCF, cross‐correlation.

### 1.2 Assessment Protocol

The participant performed two running tasks: free (overground) running and treadmill running.

For the free running task, a force plate (9287A, Kistler Japan Co. Ltd., Tokyo, Japan; 1000 Hz) was positioned along a 10 m indoor walkway, and measurements were obtained while the participant ran at a comfortable, self‐selected speed. For the treadmill task, the participant ran on a treadmill (Rehabilitation Treadmill, model 945–250; Biodex Medical Systems, Shirley, NY, USA) at four speeds (3, 4, 6, and 8 km/h) for 1 min at each speed. Lower‐limb joint moments and joint angles during running were calculated using a three‐dimensional motion capture system (Vicon Motion Systems Ltd., Oxford, UK; 50 Hz). We used the Vicon Plug‐in Gait full‐body model, attached 35 infrared markers according to the model, and conducted measurements after a sufficient familiarization period to minimize the learning effects [5, 6, 12]. Joint moments were computed only for the overground trials, where ground reaction forces (GRF) were available; treadmill analyses used kinematic signals only.

The prespecified discontinuation criteria were (1) inability to maintain the target treadmill speed despite verbal cueing (primary criterion); (2) participant request to stop; (3) signs of ischemia or acute neurological symptoms (pallor/cyanosis, dizziness, ataxia, confusion); (4) new‐onset chest pain, severe dyspnea/wheezing, or musculoskeletal pain that limits movement; and (5) dangerous gait instability (near‐fall). The test was supervised by a licensed therapist, and handrails and an emergency stop button were available as needed.

### 1.3 Data Processing and Coordination Metrics

#### 1.3.1 Marker Processing and Filtering

Marker trajectories were labeled in Vicon Nexus and low‐pass filtered at 6 Hz (fourth‐order Butterworth). The sagittal hip, knee, and ankle joint angles (°) and net moments (N·m·kg^−1^) were computed. The gait was segmented into foot‐strike (FS), mid‐stance (MS), and toe‐off (TO). These steps follow standard running analyses in able‐bodied and impaired populations [1, 3, 4, 8], noting that speed increases typically entail coupled shifts in step length and step rate, which drive coordination changes [13].

#### 1.3.2 Event Detection (Overground vs. Treadmill)

Overground trials used vertical ground reaction force (GRF) to identify events, whereas treadmill trials used kinematic heuristics (foot marker trajectory/velocity).

#### 1.3.3 Visualization of Interjoint Coordination (Lissajous Plots)

For treadmill trials, we visualized the hip–knee, hip–ankle, and knee–ankle couplings using Lissajous plots. For each speed and limb, the full 60‐s trial was plotted as continuous angle–angle trajectories without stride selection or averaging. The axes were scaled identically across speeds [14]. All figures and text have been newly created for this submission.

#### 1.3.4 Cross‐Correlation Function (CCF) for Coupling Magnitude and Timing

The hip angle was used as the reference signal. Signals were *z*‐score normalized and analyzed with an (unbiased) cross‐correlation over a ±0.30‐s lag range with a 0.02‐s resolution. For each speed × joint pair, we used a single continuous 60‐s recording to characterize continuous interjoint coordination. Representative coupling and timing were defined by the global peak correlation coefficient (*r*) and its associated lag time. By convention, negative lags indicate the hip leading the distal joint, and positive lags indicate the distal joint leading the hip. Because the design provides no independent replicates and the joint angle series exhibits serial autocorrelation, 95% confidence intervals and *p* values were not estimated.

#### 1.3.5 Lissajous‐Derived Metrics

##### 1.3.5.1 Eccentricity

For each speed and joint pair, stride‐wise joint angles were time‐normalized to 0–100% of the gait cycle and concatenated across the 60‐s trial. A PCA‐based ellipse was fitted to the pooled angle–angle data, and semi‐axes were defined from the covariance eigenvalues as $a=\sqrt{\lambda_{1}}$ and $b=\sqrt{\lambda_{2}}$. Eccentricity was computed as $\text{Eccentricity}=\sqrt{1-\frac{b^{2}}{a^{2}}}=\sqrt{1-\frac{\lambda_{2}}{\lambda_{1}}}$ (range 0–1; higher values indicate a more elongated, elliptical coordination pattern). The axes were scaled identically across speeds [14].

##### 1.3.5.2 Absolute Area

For each speed, joint pair, and side, the time‐normalized joint angles were concatenated across the 60‐s trial to form a closed angle–angle loop. The enclosed area (deg^2^)

$$A=\left|\frac{1}{2}\sum_{i=1}^{N}\left(x_{i}y_{i+1}-x_{i+1}y_{i}\right)\right|,\text{with}\;\left(x_{N+1}y_{N+1}\right)=\left(\chi_{1},y_{1}\right)$$

Session‐level values were reported for display, and the axes were scaled identically across speeds [14].

#### 1.3.6 Reporting Standard

This case report adhered to the CARE guidelines (2013) [15].

## 2 Results

### 2.1 Overground Mechanics (Free Running)

**FIGURE 1:**
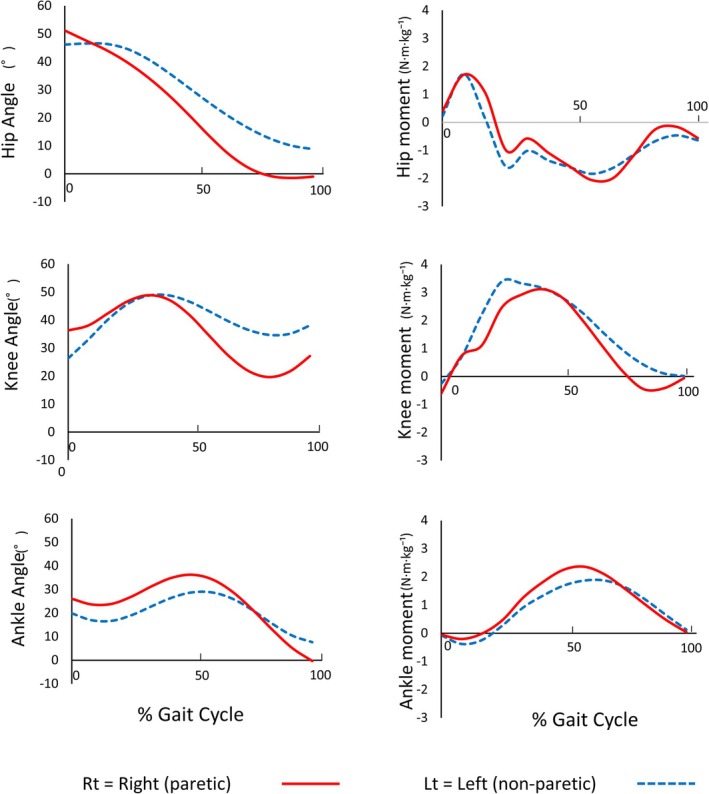
Overground running at a self‐selected speed. Sagittal joint angles and net joint moments (normalized to body mass) across the gait cycle. Red solid line = right paretic limb; blue solid line = left nonparetic limb. Percentage of gait cycle is defined from ipsilateral foot‐strike to the next foot‐strike. Note the larger hip extensor moment on the paretic side, together with persistent knee flexion bilaterally. (Units: Angle (°); Moment (N·m·kg^−1^)).

Overground joint moments are summarized in Table 2, and lower‐limb joint angles in Table 3. The paretic hip generated a larger extension moment than the nonparetic side (2.05 ± 0.32 vs. 1.49 ± 0.21 N·m·kg^−1^), whereas the nonparetic knee generated a larger extension moment than the paretic side (3.57 ± 0.24 vs. 2.79 ± 0.41 N·m·kg^−1^). At the ankle, the plantarflexion moment was slightly greater on the paretic side (2.31 ± 0.23 vs. 2.08 ± 0.22 N·m·kg^−1^). The knee remained flexed throughout the gait cycle on both limbs (peak 119.9° nonparetic vs. 98.0° paretic), and the ankle remained dorsiflexed by 0.9**°** (nonparetic) and 2.7° (paretic). At late stance, the hip angle was 4.8° in extension (nonparetic) versus 1.7° in flexion (paretic) (Figure 1, Table 3).

**TABLE 2:** Lower‐limb joint moments (overground, self‐selected speed).

Variable	Rt (paretic)		Lt (nonparetic)	
	Extension (mean ± SD)	Flexion (mean ± SD)	Extension (mean ± SD)	Flexion (mean ± SD)
Hip	2.05 ± 0.32	−2.07 ± 0.11	1.49 ± 0.21	−2.19 ± 0.34
Knee	2.79 ± 0.41	−0.68 ± 0.02	3.57 ± 0.24	−0.78 ± 0.22
Ankle	2.31 ± 0.23	−0.14 ± 0.05	2.08 ± 0.22	−0.32 ± 0.08

*Note:* Values are means ± SD from overground running at self‐selected speed. Moments are normalized to body mass (N·m·kg^−1^). Ankle: extension = plantarflexion; flexion = dorsiflexion.

Abbreviations: Lt, left (nonparetic); Rt, right (paretic).

**TABLE 3:** Lower‐limb joint angles (overground, self‐selected speed).

Variable	Rt (paretic)		Lt (nonparetic)	
	Extension (mean ± SD)	Flexion (mean ± SD)	Extension (mean ± SD)	Flexion (mean ± SD)
Hip	−1.7 ± 3.4	78.0 ± 1.4	4.8 ± 6.5	70.3 ± 1.2
Knee	−19.2 ± 5.7	98.0 ± 2.5	−19.2 ± 5.2	119.9 ± 3.9
Ankle	−2.7 ± 1.5	35.3 ± 2.4	−0.9 ± 5.5	35.1 ± 5.4

*Note:* Angles in degrees. Signs and joint conventions follow the model specified in Methods (Plug‐in‐Gait). Ankle: extension = plantarflexion; flexion = dorsiflexion.

Abbreviations: Lt, left (nonparetic); Rt, right (paretic).

### 2.2 Treadmill Multi‐Speed Coordination

**FIGURE 2:**
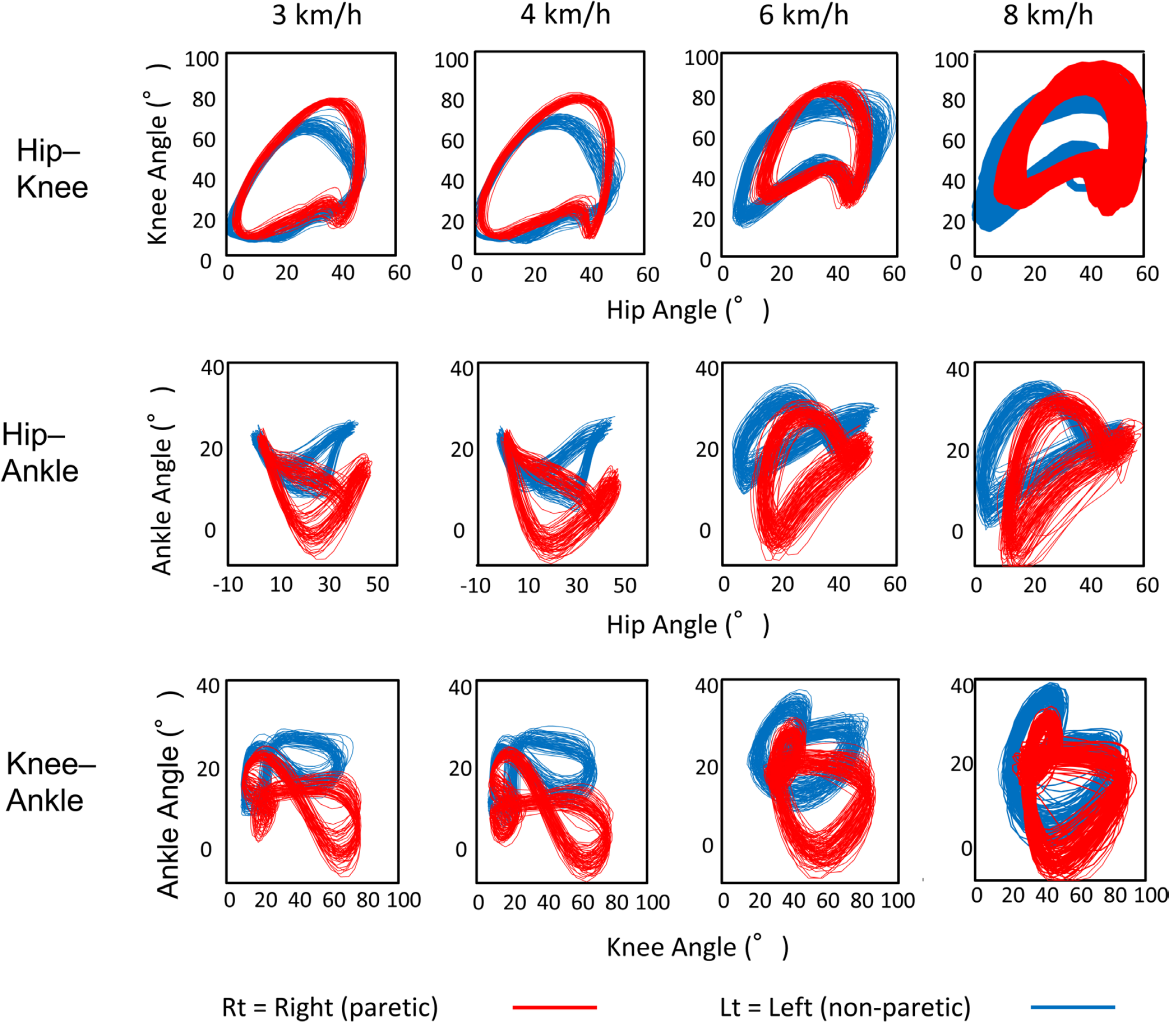
Speed‐dependent interjoint coordination during treadmill running. Angle–angle (Lissajous) plots for the hip–knee, hip–ankle, and knee–ankle pairs at 3, 4, 6, and 8 km/h. Angles in degrees; axes were scaled identically across speeds. Red = right paretic limb; blue = left nonparetic limb.

Lissajous plots (Figure 2) showed that hip–knee coordination was relatively stable across speeds, and hip–ankle and knee–ankle couplings changed more with speed, especially at 6–8 km/h. On the paretic side, the loop shapes remained uniform/rigid with speed; on the nonparetic side, they became increasingly complex, indicating adaptive coupling.

### 2.3 Cross‐Correlation Function (CCF)

The detailed coefficients and lags are listed in Table 4. For hip–knee, r on the nonparetic side decreased with speed from 3 to 8 km/h (0.46 to 0.18), whereas the paretic side remained approximately constant (0.52–0.54). For the hip–ankle, the non‐paretic side transitioned from −0.46 to 0.56, whereas the paretic side remained consistently positive (0.25–0.55). The time lags indicated that the hip led the knee by approximately 0.10–0.16 s across speeds on both sides. For the hip–ankle, the lag shifted with speed; the hip led at 3–4 km/h (−0.08 to −0.22 s), but the ankle led slightly at 6–8 km/h (+0.06 to +0.10 s) in both limbs.

**TABLE 4:** Correlation coefficients and time lags (s) between the hip and distal joints during treadmill running, obtained using cross‐correlation analysis.

		Correlation coefficient (*r*)		Time lag (s)	
		Rt	Lt	Rt	Lt
Hip joint vs. Knee joint	3 km/h	0.52	0.46	−0.16	−0.16
	4 km/h	0.43	0.34	−0.16	−0.16
	6 km/h	0.54	0.23	−0.10	−0.12
	8 km/h	0.50	0.18	−0.10	−0.14
Hip joint vs. Ankle joint	3 km/h	0.34	−0.46	−0.20	−0.08
	4 km/h	0.25	−0.52	−0.22	−0.10
	6 km/h	0.55	0.40	0.06	0.10
	8 km/h	0.46	0.56	0.08	0.06

*Note:* CCF was computed using the hip angle as the reference. Lag (s): negative = hip leading; positive = distal leading. *r* = Pearson's correlation coefficient.

Abbreviations: Lt, left (nonparetic); Rt, right (paretic).

### 2.4 Lissajous Figures

**FIGURE 3:**
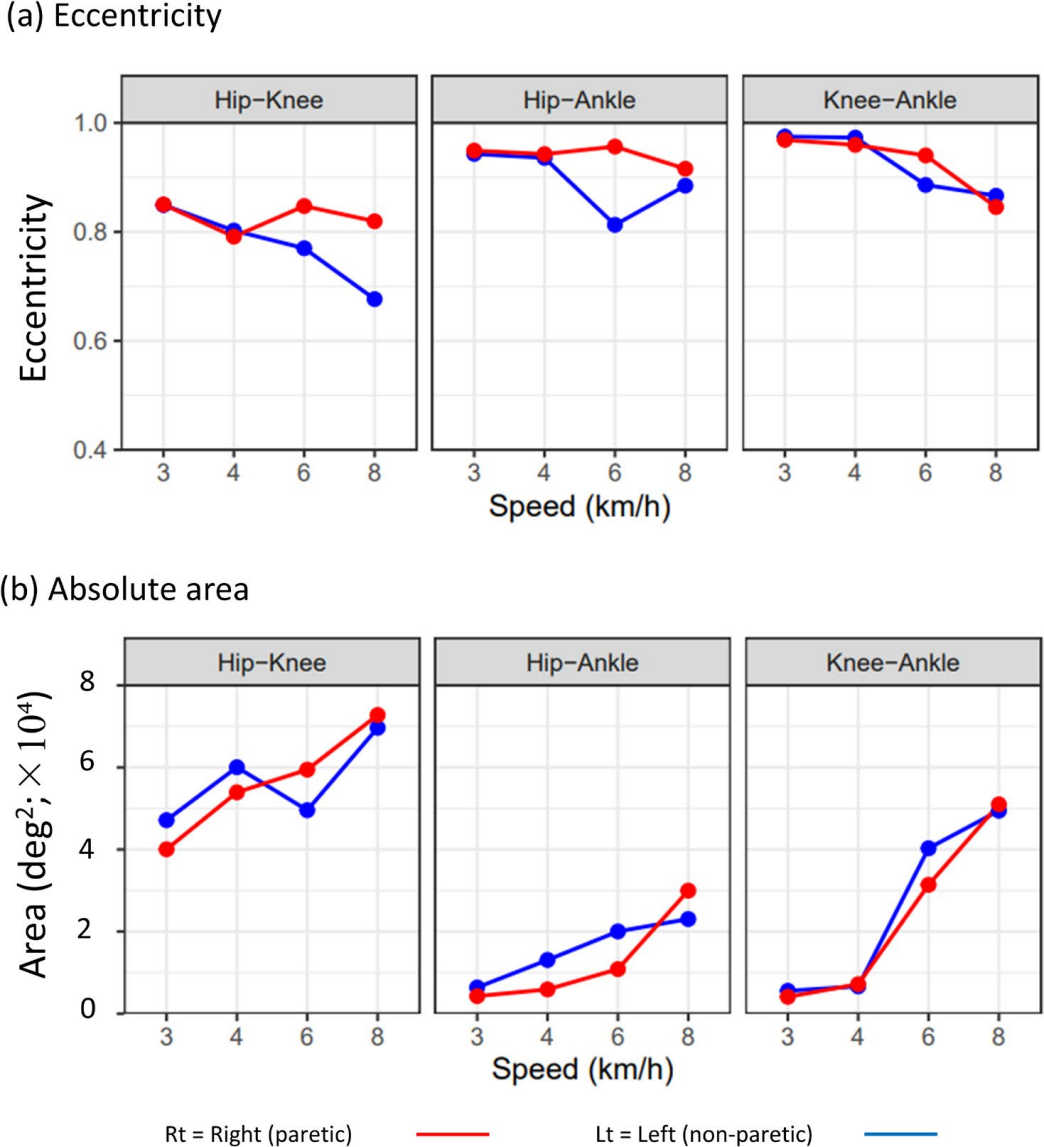
(a) Eccentricity (dimensionless; 0–1 scale). (b) Absolute area (deg^2^; ×10^4^). Red = right (paretic); blue = left (non‐paretic). Speeds: 3, 4, 6, and 8 km/h. The axes were scaled identically across speeds.

Figure 3 shows the changes in eccentricity and absolute area as a function of treadmill speed (3, 4, 6, and 8 km/h).

#### 2.4.1 Eccentricity

The eccentricity values were generally high (0.80–0.95), indicating predominantly elliptical coordination. In the hip–knee pair, eccentricity decreased slightly as speed increased, suggesting a shift toward a more linear coordination pattern, particularly on the paretic side. The hip–ankle and knee–ankle pairs showed only minor speed‐dependent changes in eccentricity.

#### 2.4.2 Absolute Area

The enclosed area increased with treadmill speed, particularly for the hip–knee and knee–ankle pairs, reflecting greater variability and amplitude of interjoint motion at higher speeds. The hip–ankle pair exhibited less speed‐dependent changes.

## 3 Discussion

This case demonstrates a coherent compensatory profile that permits running despite chronic hemiplegia, including proximal dominance at the paretic hip, limited knee extension, sustained ankle dorsiflexion, and rigid distal coordination that resists speed‐driven reorganization. The nonparetic limb exhibits speed‐dependent adaptability, consistent with the previously described “expected and novel” compensatory contributions of the intact side in hemiparetic gait [16]. From a mechanical standpoint, reduced plantarflexor contribution shifts propulsive demands proximally, increasing hip extensor workload and potentially predisposing to soft tissue overuse, an issue recognized broadly in running biomechanics [3, 4, 5, 7, 8, 17, 18].

In relation to prior studies, our observation that the ankle leads the hip at ≥ 6 km/h complements prior evidence of distal‐to‐proximal redistribution and reduced distal propulsion in post‐stroke locomotion [19, 20]. In this context, a speed‐evoked coupling reversal is plausible: as belt speed escalates and push‐off demands increase, timing may shift toward a distal lead to generate propulsion, whereas stiff‐knee phenomena and altered joint stiffness regulation can further perturb inter‐joint timing [21]. Whereas much of the literature emphasizes overground patterns, our treadmill multi‐speed protocol shows that such timing reversals can emerge with speed escalation, supporting the view that task demands (speed and environmental constraints) shape coordination [22]. This interpretation aligns with athletic/parasport reports and adaptation studies demonstrating speed‐ and task‐dependent reorganization of spatiotemporal control after stroke [10, 23]. Together, these data indicate that speed‐specific testing is necessary to expose latent timing deficits that may be missed at comfortable speeds but are directly relevant to running reintegration and targeted rehabilitation, focusing on distal propulsion and inter‐joint timing.

### 3.1 Clinical Interpretation of Lissajous‐Derived Coordination Metrics in Post‐Stroke Gait

This study demonstrates that Lissajous‐based metrics can capture distinct alterations in inter‐joint coordination in a post‐stroke individual with right hemiparesis. Regarding eccentricity, the reduction observed in the hip–knee pair with increasing speed indicates a shift toward more linear, constrained coordination on the paretic side [14, 24], likely reflecting compensatory reliance on hip flexion to overcome limited knee flexion during the swing phase, consistent with clinical observations such as hip hiking or forward leg thrust in hemiparetic gait [21]. Additionally, the increase in the Lissajous absolute area at higher speeds suggests increasing variability and instability of coordination [14, 24], and on the paretic side, this pattern is consistent with reduced neuromuscular control and overreliance on the nonparetic limb [19, 20] to attain higher treadmill speeds.

### 3.2 Clinical Implications (Actionable Points)

In post‐stroke hemiparesis, increasing treadmill speed does not simply scale existing coordination; it tends to amplify asymmetry and instability, particularly in the knee–ankle coupling. Accordingly, interventions should prioritize distal control and inter‐joint timing to support more efficient and symmetric gait at higher speeds.

– *Distal propulsion*. Emphasizing late‐stance knee extension and ankle plantarflexion to redistribute propulsive work distally; using progressive plantarflexor strengthening, task‐specific push‐off drills, and cueing to limit persistent knee flexion.

– *Interjoint timing*. Train timing plasticity with metronome/visual feedback, phase‐specific prompts, and variable‐speed treadmill paradigms to loosen rigid distal coupling on the paretic side and promote flexible hip–ankle lags (approximately −0.22 s at 3–4 km/h to approximately +0.10 s at 6–8 km/h; see Table 4).

– *Implementation considerations*. Because this protocol was implemented in a laboratory, real‐world deployment may be constrained by equipment availability (e.g., treadmill, safety harness, real‐time feedback). Where unavailable, core elements can be approximated with low‐tech substitutes (metronome cues, visual floor markers, simple heel‐rise/step‐to‐push‐off drills). A second barrier is that not all poststroke patients can run or tolerate higher belt speeds; for these individuals, a graded progression from fast walking to jogging with speed ceilings and brief intervals can target the same timing and distal propulsion goals. These pragmatic adaptations preserve the clinical intent while acknowledging resource and recovery constraints.

– *Neuroplasticity perspective*. These targets operationalize key principles of neuroplasticity—task specificity, repetition/intensity, and timing‐dependent learning—via speed‐graded treadmill practice, phase‐specific cues, and timing feedback to reshape interjoint coordination. Although a single session cannot demonstrate long‐term change, these mechanisms are consistent with pathways supporting durable performance gains after severe injury (e.g., stroke, spinal cord injury), and longitudinal follow‐ups are needed to confirm transfer and retention. Consistent with the established principles of experience‐dependent plasticity—repetition/intensity and task‐specific practice—we frame timing‐focused and distal‐strengthening interventions as drivers of adaptive reorganization after stroke [25, 26, 27]. These recommendations align with contemporary neurorehabilitation frameworks that synthesize motor learning mechanisms with brain plasticity [27].

## 4 Limitations and Future Directions

This is a single‐case, single‐session study; therefore, the findings are inherently limited in their generalizability, and population‐level inference is not intended. In addition, the absence of a control cohort (e.g., level‐matched post‐stroke or able‐bodied runners) limits our ability to contextualize the findings. No post‐intervention follow‐up outcomes were available, precluding inferences about the durability or trainability of the observed patterns. Moreover, testing was conducted in a controlled laboratory setting on a treadmill, which may limit the ecological validity relative to overground or field conditions. Finally, because electromyography (EMG) and metabolic measurements were not acquired, neuromuscular strategies and energetic costs underlying the observed compensations cannot be directly inferred.

To address these constraints, future studies should validate the present observations in larger samples and different populations using harmonized, speed‐matched protocols and incorporate level‐matched comparative cohorts to provide benchmarks for interjoint coupling and timing. Longitudinal designs with postintervention follow‐ups are needed to test durability and transferability. Real‐world/overground assessments (e.g., with wearable sensors) would improve ecological validity, and adding surface EMG (e.g., plantarflexor timing, co‐contraction indices) and basic metabolic indices would help link coordination patterns to neuromuscular and energetic demands.

### 4.1 Patient Perspective

The athlete reported valuing objective feedback that linked specific joint patterns (hip–dominant push, persistent knee flexion) with fatigue and speed plateaus. He expressed a willingness to engage in timing‐focused treadmill work and ankle‐centric strengthening to pursue competitive goals (Paraphrased for anonymity).

## 5 Conclusion

This case underscores that running after stroke can be sustained via a proximal‐dominant, rigid‐distal strategy; however, such compensation limits speed‐dependent adaptability. Quantifying Lissajous‐based coupling and CCF lags revealed a clear intervention target: enhancing distal output and timing plasticity to rebalance load and improve efficiency. These insights provide practice‐ready guidance for clinicians training high‐functioning stroke survivors toward safe and efficient running.

## Author Contributions

**Noboru Chiba:** conceptualization, formal analysis, funding acquisition, investigation, methodology, project administration, visualization, writing – original draft, writing – review and editing. **Tadayoshi Minamisawa:** investigation, methodology, resources, software, visualization, writing – review and editing.

## Ethics Statement

Ethics approval was obtained.

## Consent

Written informed consent for participation and publication was obtained from the participant. This study was approved by the Ethics Committee of Yamagata Prefectural University of Health Sciences (Approval no. 1501‐14). This study conformed to the Declaration of Helsinki (2013 revision).

## Conflicts of Interest

The authors declare no conflicts of interest.

## Data Availability

De‐identified time‐series data sets and analysis code (Lissajous/CCF) will be made publicly available upon acceptance in an open repository (OSF or Zenodo), and the DOI will be added to the final article. During peer review, an anonymized, view‐only link can be provided to editors/reviewers upon request.
